# Impact of Total Epinephrine Dose on Long Term Neurological Outcome for Cardiac Arrest Patients: A Cohort Study

**DOI:** 10.3389/fphar.2021.580234

**Published:** 2021-05-28

**Authors:** Xiaowei Shi, Jiong Yu, Qiaoling Pan, Yuanqiang Lu, Lanjuan Li, Hongcui Cao

**Affiliations:** ^1^ State Key Laboratory for Diagnosis and Treatment of Infectious Diseases, National Clinical Research Center for Infectious Diseases, Collaborative Innovation Center for Diagnosis and Treatment of Infectious Diseases, The First Affiliated Hospital, Zhejiang University School of Medicine, Hangzhou, China; ^2^ Division of Hepatobiliary and Pancreatic Surgery, Department of Surgery, The First Affiliated Hospital, Zhejiang University School of Medicine, Hangzhou, China; ^3^ NHFPC Key Laboratory of Combined Multi-organ Transplantation, Hangzhou, China; ^4^ Department of Emergency Medicine, The First Affiliated Hospital, Zhejiang University School of Medicine, Hangzhou, China; ^5^ Zhejiang Provincial Key Laboratory for Diagnosis and Treatment of Aging and Physic-chemical Injury Diseases, Hangzhou, China

**Keywords:** epinephrine dose, neurological outcome, cardiac arrest patients, cohort study, multivariate analysis

## Abstract

**Introduction:** Although epinephrine is universally acknowledged to increase return of spontaneous circulation (ROSC) after cardiac arrest, its balanced effects on later outcomes remain uncertain, causing potential harm during post-resuscitation phase. Recent studies have questioned the efficacy and potential deleterious effects of epinephrine on long-term survival and neurological outcomes, despite that the adverse relationship between epinephrine dose and outcome can be partially biased by longer CPR duration and underlying comorbidities. This study explored the long-term effect of epinephrine when used in a cohort of patients that underwent cardiac arrest during cardiopulmonary resuscitation.

**Methods:** The data were originally collected from a retrospective institutional database from January 2007 to December 2015 and are now available on Dryad (via: https://doi.org/10.5061/dryad.qv6fp83). Use of epinephrine was coded by dose (<2 mg, 2 mg, 3–4 mg, ≥5 mg). A favorable neurological outcome was defined using a Cerebral Performance Category (CPC) 1 or 2. The association between epinephrine dosing and 3-months neurological outcome was analyzed by univariate analysis and multivariate logistic regression.

**Results:** Univariate and multivariate analysis demonstrated a negative association between total epinephrine dose and neurological outcome. Of the 373 eligible patients, 92 received less than 2 mg of epinephrine, 60 received 2 mg, 97 received 3–4 mg and 124 received more than 5 mg. Compared to patients who received less than 2 mg of epinephrine, the adjusted odds ratio (OR) of a favorable neurological outcome was 0.8 (95% confidence interval [CI]: 0.38–1.68) for 2 mg of epinephrine, 0.43 (95% confidence interval [CI]: 0.21–0.89) for 3–4 mg of epinephrine and 0.40 (95% confidence interval [CI]: 0.17–0.96) for more than 5 mg of epinephrine.

**Conclusion:** In this cohort of patients who achieved ROSC, total epinephrine dosing during resuscitation was associated with a worse neurological outcome three months after cardiac arrest, after adjusting other confounding factors. Further researches are needed to investigate the long-term effect of epinephrine on cardiac arrest patients.

## Introduction

Standard-dose epinephrine for adult cardiac arrest is defined as 1 mg given intravenously every 3–5 min until return of spontaneous circulation (ROSC) regardless of cardiac arrest rhythm (Bossaert et al., 2015) by current American Heart Association and European Resuscitation Council guidelines. Epinephrine can effectively increase aortic blood pressure via its alpha-adrenergic vasopressor activity, which contributes to coronary perfusion and subsequently helps achieve ROSC during chest compression (Link et al., 2015). On the other hand, adverse effects including impaired cerebral microvascular flow (Ristagno et al., 2009) and myocardial depression (Angelos et al., 2008) are observed in laboratory. Likewise, epinephrine dosing is also associated with coagulation (Larsson et al., 1989), impaired tissue oxygen utilization and lactate clearance (Rivers et al., 1994) in humans.

For the past few decade, as resuscitation interventions have become more successful, there is an increasing need to reconsider the patient-centered outcomes such as functional status and quality of life in addition to returning of pulses (Becker et al., 2011). Although epinephrine is associated with a greater likelihood of ROSC, this early potential benefit for the heart doesn’t guarantee good patient outcomes, as the vast majority of patients resuscitated from cardiac arrest present in coma or with altered level of consciousness (Geocadin et al., 2019). Recent studies have questioned the efficacy and potential deleterious effects of epinephrine on long-term survival and neurological outcomes (Ong et al., 2007; Mayor, 2014).

In a large observational study of OHCA patients in Japan, prehospital epinephrine administration was significantly associated with increased chance of ROSC before hospital arrival but decreased likelihood of survival and worse functional status one month after the event (Hagihara et al., 2012). Admittedly, the total dose of epinephrine administered is proportional to how long a patient remains in cardiac arrest, resulting in higher doses for patients who fail to respond to initial treatment (Callaway, 2013). Therefore, adverse relationship between epinephrine dose and outcome can be partly attributed to “resuscitation time bias” and underlying comorbidities (Matos et al., 2013). Further work from a randomized clinical trial demonstrated that more survivors had severe neurological impairment in the epinephrine-treated group (Perkins et al., 2018), although the between-group difference in the percentage of a favorable neurological outcome at hospital discharge was not statistically significant when compared to placebo group.

We sought to explore the long-term effect of epinephrine when used in cardiopulmonary resuscitation. In this secondary analysis, we described the association between epinephrine dosing during cardiac arrest and 3-months neurological functions among a cohort of patients that underwent cardiac arrest.

## Methods

### Study Population

This is a secondary analysis of a retrospective study (Iesu et al., 2018) where the dataset was collected by Iesu et al. and is now available on Dryad (via: https://doi.org/10.5061/dryad.qv6fp83). The former study was originally performed in the Department of Intensive Care at Erasme Hospital, Brussels (Belgium) and was approved by the local Ethical Committee (Comite´ d’Ethique Hospitalo-Facultaire Erasme-ULB) while waiving the need for informed consent considering its retrospective nature. In the original study, the data were collected from a retrospective institutional database (January 2007 to December 2015), where patients admitted after in-hospital cardiac arrest (IHCA) and out-of-hospital cardiac arrest (OHCA) with a Glasgow Coma Scale (GCS) < 9 were included. Exclusion criteria were missing data on liver function or death less than 24 h after ICU admission. All patients were treated with therapeutic hypothermia, targeting a body temperature between 32 and 34°C for 24 h, according to a standardized institutional post-resuscitation management protocol that has been extensively described elsewhere (Tujjar et al., 2015; Iesu et al., 2018).

### Data Collection

In the original study, Iesu et al. collected data on demographics, comorbidities (including diabetes, hypertension, chronic renal failure, chronic heart failure, and previous neurological diseases) and first aid information [bystander CPR, time to ROSC (the arrival of emergency medical care), total epinephrine dose and non-shockable rhythm] in all patients. Lactate and glucose level on admission, shock, the length of ICU stay, ICU death and hospital death was recorded, as with the proportion of IHCA and OHCA. Information about whether patients underwent ECPR, quality or duration of CPR was not explicitly stated in the original database or manuscript. Aiming to analyze the association between epinephrine dosage and neurological functions of survivors, cerebral performance categories score (CPC) was employed from the original study to assess neurological outcomes three months after cardiac arrest (1 = no or mild neurological disability, 2 = moderate neurological disability, 3 = severe neurological impairment, 4 = vegetative state, 5 = death). The neurological outcome was defined as favourable with CPC 1–2 and unfavourable with CPC 3–5 (Jennett and Bond, 1975). The CPC evaluation was performed during follow-up visits or by telephone interview with the general practitioner.

### Statistical Analysis

All the analyses were performed using the statistical software packages R (http://www.R-project.org, The R Foundation) and EmpowerStats (http://www.empowerstats.com, X&Y Solutions, Inc., Boston, MA). *p*-value < 0.05 (two-tailed) was considered statistically significant.

In descriptive statistics, continuous variables were presented as mean ± standard deviation (normal distribution) or median [Q1-Q3] (skewed distribution). Classified variables were presented as number and percentage. Chi-square test (for categorical variables), One-Way ANOVA test (for continuous variables with normal distribution), or Mann-Whitney U test (for continuous variables with skewed distribution) were employed to calculate the significance among different epinephrine dosage groups.

First, univariate analysis was conducted to investigate the correlations between different factors (age, gender, epinephrine dosage, etc) and neurological status three months after cardiac arrest. Second, multivariate linear regression was employed to calculate the independent effect of epinephrine dose on neurological outcome. In this step, three adjust models were employed: 1) model 1: no covariates were adjusted; 2) model 2: only adjusted for age and sex; 3) model 3: specific covariates were adjusted as potential confounders if they change the estimates of epinephrine dosage (X) on neurological outcome three months after cardiac arrest (Y) by more than 10% or significantly associated with the neurological outcome (Y). The following covariates were selected a priori on the basis of established associations and/or plausible biological relations and tested: age, gender, chronic renal failure, previous neurological disease, OHCA, witness arrest, bystander CPR, time to ROSC, non-shockable rhythm, baseline lactate, baseline glucose, TTM, ICU length of stay.

Coefficient of each variable in univariate and multivariate model was presented in Supplementary Appendix Table A1. Assumption check for multivariate logistic regression model was presented in Supplementary Appendix Table A2. Specifically, age and baseline lactate were found curvilinear, and were further checked by generalized addictive model for their effect value. The detailed information were listed in Supplementary Appendix Table A3.

## Results

**FIGURE 1:**
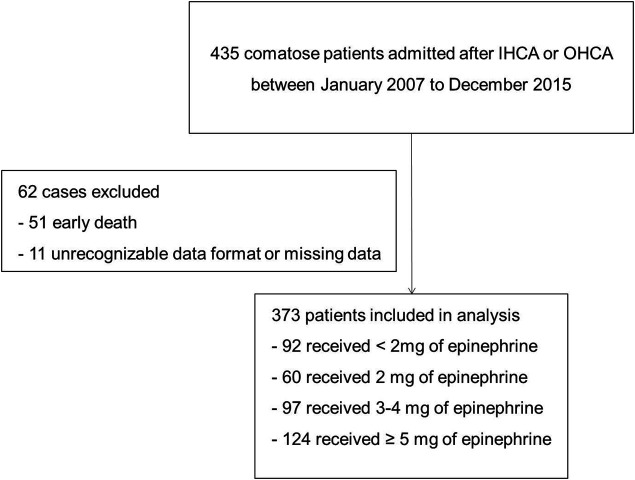
Flowchart of patient selection. Abbreviation: IHCA, in-hospital cardiac arrest; OHCA: out-of-hospital cardiac arrest.

From 435 patients in total, 61 of those were excluded for the reason of early death (*n* = 51) or missing data on liver transaminases, coagulation or total bilirubin (*n* = 10), according to Iesu et al. (Iesu et al., 2018); additionally, in this manuscript, 1 patient was excluded because of unrecognizable data format in Excel downloaded from Dryad. 373 patients were included in the final analysis based on the inclusion and exclusion criteria (Figure 1).

### Baseline Characteristics of Selected Participants

Descriptive statistics of the study population were provided in Table 1. Generally, the cohort was 61.8 ± 15.4 years of age, and 72.1% of them were male. 61.0% of the patients had OHCA, 85.5% had a witnessed CA, 59.0% had a non-shockable rhythm, and 14.5% had pre-existing neurological diseases. The ICU length of stay was 4 [2–9] days, 55.6% patients died in hospital and 39.7% patients had a favourable neurological outcome.

**TABLE 1:** Clinical characteristics of patients at the time of hospital admission.

Variable	Statistics (N = 373)	Epinephrine dosage groups				
		<2 mg (N = 92)	2 mg (N = 60)	3–4 mg (N = 97)	≥5 mg (N = 124)	*p* value ^a^
Age (year)	61.8 ± 15.4	65.47 ± 15.09	62.60± (16.38)	61.92 ±(14.95)	58.62 ±(14.98)	0.013
Gender(male)	269 (72.1%)	63 (68.48%)	40 (66.67%)	78 (80.41%)	88 (70.97%)	0.180
Diabetes	90 (24.1%)	24 (26.09%)	17 (28.33%)	27 (27.84%)	22 (17.74%)	0.234
Hypertension	159 (42.6%)	46 (50.00%)	27 (45.00%)	38 (39.18%)	48 (38.71%)	0.329
Previous neurological disease	54 (14.5%)	19 (20.65%)	10 (16.67%)	15 (15.46%)	10 (8.06%)	0.064
Chronic renal failure	62 (16.6%)	18 (19.57%)	9 (15.00%)	17 (17.53%)	18 (14.52%)	0.767
Chronic heart failure	78 (20.9%)	16 (17.39%)	12 (20.00%)	17 (17.53%)	33 (26.61%)	0.281
OHCA	180 (61.0%)	45 (48.91%)	25 (41.67%)	58 (59.79%)	79 (64.23%)	0.013
Witnessed arrest	319 (85.5%)	85 (92.39%)	55 (91.67%)	85 (87.63%)	94 (75.81%)	0.002
Bystander CPR	253 (67.8%)	78 (84.78%)	44 (73.33%)	63 (64.95%)	68 (54.84%)	<0.001
Time to ROSC (min)	18.1 ± 14.1	7.28 ± 6.25	10.30 ± 6.33	17.26 ± 10.40	30.51 ± 13.95	<0.001
Non-shockable rhythm	220 (59.0%)	56 (60.87%)	35 (58.33%)	51 (52.58%)	78 (62.90%)	0.461
Baseline lactate (mEq l^−1^)	6.3 ± 3.3	6.35 ± 3.68	5.67 ± 2.65	6.43 ± 3.66	6.32 ± 3.05	0.525
Baseline glucose (mg dl^−1^)	234.8 ± 125.3	254.95 ± 153.64	225.73 ± 114.15	227.62 ± 116.06	229.86 ± 113.18	0.362
TTM	78 (84.78%)	49 (81.67%)	93 (95.88%)	111 (89.52%)	78 (84.78%)	0.024
ICU stay (day)	7.8 ± 9.7	7.41 ± 8.44	7.83 ± 8.21	7.97 ± 9.05	8.38 ± 11.69	0.913
ICU death	153 (51.9%)	36 (39.13%)	23 (38.33%)	55 (56.70%)	80 (64.52%)	<0.001
Hospital death	164 (55.6%)	40 (43.48%)	27 (45.00%)	59 (60.82%)	86 (69.35%)	<0.001
Favorable neurological outcomes	148 (39.7%)	50 (54.35%)	30 (50.00%)	34 (35.05%)	34 (27.42%)	<0.001

Abbreviations: CPR, cardiopulmonary resuscitation; ICU, intensive care unit; OHCA, out-of-hospital cardiac arrest; ROSC, restoration of spontaneous circulation; TTM, targeted temperature management.

^a^ *p* value is calculated as a result of group comparison among epinephrine dosage groups.

### Univariate Analysis

We reclassified the baseline data according to neurological status before conducting univariate analysis and the overall results were presented in Table 2. Compared to those with unfavorable neurological outcomes, patients with favorable neurological outcomes were younger, had shorter time to ROSC and less total epinephrine dose, and were less frequently to suffer from previous neurological disease or shock during hospital stay, while they were more likely to experience a witnessed CA, a bystander CPR and a shockable rhythm.

**TABLE 2:** Univariate analysis for factors and their association with neurological outcomes three months after cardiac arrest.

Variable	Unfavorable neurological outcomes (n = 225)	Favorable neurological outcomes (n = 148)	Univariate analysis (odds ratio, 95% CI)	*p* value
Age (year)	63.64 ± 15.96	59.03 ± 14.09	0.98 (0.97, 0.99)	0.005
Gender (male)	160 (71.11%)	109 (73.65%)	1.13 (0.71, 1.80)	0.611
Hypertension	94 (41.78%)	65 (43.92%)	1.10 (0.72, 1.67)	0.656
Diabetes	60 (26.67%)	30 (20.27%)	0.69 (0.42, 1.13)	0.14
Chronic heart failure	50 (22.22%)	28 (18.92%)	0.82 (0.49, 1.38)	0.456
Chronic renal failure	41 (18.22%)	21 (14.19%)	0.74 (0.42, 1.32)	0.307
Previous neurological disease	41 (18.22%)	13 (8.78%)	0.43 (0.22, 0.84)	0.014
OHCA	125 (55.80%)	82 (55.41%)	0.99 (0.65, 1.51)	0.977
Bystander CPR	139 (61.78%)	114 (77.03%)	2.06 (1.29, 3.29)	0.003
Witnessed arrest	183 (81.33%)	136 (91.89%)	2.59 (1.31, 5.10)	0.006
Time to ROSC (min)	20.09 ± 14.86	15.03 ± 12.25	0.97 (0.96, 0.99)	0.001
Epinephrine dosage (mg)	4.75 ± 4.01	3.03 ± 2.84	0.86 (0.80, 0.92)	<0.001
Non-shockable rhythm	159 (70.67%)	61 (41.22%)	0.29 (0.19, 0.45)	<0.001
Baseline lactate (mEq l^−1^)	6.42 ± 3.46	6.00 ± 3.10	0.96 (0.90, 1.03)	0.238
Baseline glucose (mg dl^−1^)	222.22 ± 105.08	253.93 ± 149.22	1.002 (1.000, 1.004)	0.02
TTM	205 (91.11%)	126 (85.14%)	0.56 (0.29, 1.06)	0.08
ICU stay (day)	7.00 ± 10.45	9.39 ± 8.34	1.03 (1.00, 1.05)	0.026

Employing univariate linear regression, we found that previous neurological diseases, witnessed CA, bystander CPR, non-shockable rhythm, total epinephrine dosing, shock during ICU stay and baseline glucose were associated with neurological outcomes three months after CA. Among them, previous neurological diseases, total epinephrine dosage, and shock were negatively associated with neurological outcomes, while witnessed CA, shockable rhythm, bystander CPR and baseline glucose were positively correlated with neurological outcomes.

### Multivariate Regression Analysis

Aiming to calculate the independent effect of epinephrine dosage on neurological outcomes, three models were constructed based on multivariate logistic regression, with their effect values (Odds ratio, OR) and 95% confidence intervals (CI) listed in Table 3. The model-based effect value can be interpreted as how epinephrine dosage changes the likelihood of favorable neurological outcomes. For instance, in unadjusted model, the effect value for 2 mg epinephrine dosing group is 0.84, implying that compared to patients administrated with less than 2 mg of epinephrine, the likelihood of those administrated with 2 mg of epinephrine achieving favorable neurological outcomes decreases 16%.

**TABLE 3:** Logistic regression analysis for the association between total epinephrine dosage and neurological outcomes.

Epinephrine dosage (mg)	Favorable neurological outcomes (odds ratio, 95% CI, *p* value)		
	Non-adjusted	Adjust I	Adjust II
<2 mg	1.0	1.0	1.0
2 mg	0.84 (0.44, 1.61) 0.600	0.77 (0.40, 1.51) 0.452	0.80 (0.38, 1.68) 0.561
3–4 mg	0.45 (0.25, 0.81) 0.008	0.38 (0.21, 0.70) 0.002	0.43 (0.21, 0.89) 0.024
≥5 mg	0.32 (0.18, 0.56) <0.001	0.25 (0.14, 0.45) <0.001	0.40 (0.17, 0.96) 0.041

No confounding factors were adjusted in non-adjusted model. Age and gender were adjusted in Adjust I model. Age, gender, chronic renal failure, previous neurological disease, OHCA, witness arrest, bystander CPR, time to ROSC, non-shockable rhythm, baseline lactate, baseline glucose, TTM, ICU stay days were adjusted in Adjust II model.

However, unadjusted model is limited due to its univariate nature, and other factors that simultaneously impact neurological prognosis after CA must be taken into consideration. Specifically, in this manuscript, time to ROSC (min) and age were statistically significant among different epinephrine dosing groups. The former can be easily interpreted from a medical perspective, as patients with longer time to ROSC are treated with more epinephrine shots. Two more models were provided after adjusting different confounding factors, as presented in Table 3. In the fully adjusted model (model II), the results indicate that the patients who received more than 5 mg of epinephrine were 60% less likely to achieve a favorable neurological outcome than those administered <2 mg as measured by Cerebral Performance Category (CPC). Moreover, neurological outcomes were not significantly different in patients who received <2 mg of epinephrine vs. those received 2 mg. Further, the effect of epinephrine dosage on 3-months neurological outcomes is consistent among OHCA/IHCA groups and shockable/non-shockable rhythm groups, as revealed by stratification analysis in Supplementary Appendix Table A4.

## Discussion

In this secondary analysis, total epinephrine dosing during resuscitation was associated with a worse neurological outcome three months after OHCA or IHCA, after adjusting other confounding factors.

Although epinephrine is universally acknowledged to increase ROSC after cardiac arrest, its balanced effects on later outcomes remain uncertain, with potential harm during post-resuscitation phase. In a large observational study by Hagihara et al., the chance of achieving 1-month survival and favorable neurological outcomes (defined as a CPC of 1–2) were remarkably reduced in epinephrine-treated group (Hagihara et al., 2012). In another large cohort of patients who achieved ROSC, the adverse association of prehospital epinephrine administration and chance of survival was observed regardless of resuscitation duration or in-hospital interventions performed (Dumas et al., 2014). Data from randomized clinical trials have also failed to provide a conducive effect of epinephrine on longer-term outcomes. Olasveengen et al. have concluded that CPC score at discharge and 1-year survival were not significantly improved in patients that received intravenous administration of epinephrine (Olasveengen et al., 2009). Perkins et al. demonstrated that survivors from epinephrine group were more likely to display severe neurologic impairment (defined as a score of 4 or 5 on the modified Rankin scale) compared to the placebo group (Perkins et al., 2018).

These paradoxical phenomenons may be related to epinephrine’s mechanism of action. By activating α-adrenergic receptors, epinephrine robustly augments coronary perfusion during CPR to increase the likelihood of ROSC. However, at the same time, the blood flow to all other organs, including cerebrum, is reduced to support this temporary benefit for coronary perfusion. This effect may even persist after the return of pulses and eventually incur a metabolic debt from the body and brain that are detrimental to long-term outcomes despite the improvement in ROSC(Sigal et al., 2019). Moreover, the clinical effect of epinephrine is likely to hinge on timing, dosing, and patients’ conditions. Compared with previous work, our study has adjusted the effect of baseline glucose, baseline lactate, previous neurological diseases, chronic heart failure, chronic renal failure, coronary artery diseases or other confounding factors that would interfere with long-term neurological outcomes, improving accuracy and robustness of the adverse association between epinephrine dosage and neurological outcomes.

In this study, both OHCA and IHCA patients were included but the final analysis revealed no differences in outcomes between OHCA and IHCA patients. Theoretically, IHCA patients would receive more timely emergency treatments compared to OHCA patients, and may consequently achieve a better outcome; We speculated that it may be due to the OHCA group included were of a better physical condition, as they were younger (even though not statistically significant) and had fewer comorbidities such as hypertension, diabetes, chronic heart failure and chronic renal failure (see Supplementary Appendix Table A5).

For the past few decades, induced hypothermia and integrated plans of care have successfully improved the survival to hospital discharge among cardiac arrest patients (Callaway, 2012). One hypothesis is that these post-resuscitation interventions help alleviate the potential damage caused by epinephrine administration. These practices have raised expectations beyond restoration of spontaneous circulation but also functional status and quality of life after discharge (Callaway, 2012). In this study, we can’t conclude that TTM is significant associated with 3-months outcome based on the *p* value in univariate analysis. Further, in the revised Adjust II model, TTM remained as a potential confounder in evaluating the association between epinephrine dosage and 3-months neurological outcome, indicating that the negative correlation between epinephrine dosage and 3-months neurological outcome was reserved in spite of TTM intervention.

These findings should inspire further investigation on the most appropriate scheme of treatment rather than incriminating epinephrine itself. The standard 1-mg dose of epinephrine for CPR was initially defined basing on dog models (Redding and Pearson, 1962; Redding and Pearson, 1968) and has been applied on adult patients without weight or interspecies adjustment ever since. In a recent study, lower-dose epinephrine administration was not associated with OHCA outcomes in terms of survival to hospital discharge and favorable neurological status (Fisk et al., 2018). On the other hand, our results indicate that the patients who received less than 2 mg of epinephrine were more likely to present a favorable neurological outcome than those administered >5 mg as measured by Cerebral Performance Category (CPC). In addition, neurological outcomes were not significantly different in patients who received <2 mg of epinephrine vs. those received 2 mg. Similar conclusions were also reached in a large observational study using data from Penn Alliance for Therapeutic Hypothermia (PATH) registry (Larsson et al., 1989). Generally, prolonged resuscitation is accompanied by repeated and increased doses of epinephrine, but this continued administration seems detrimental to functional neurological status.

Strengths of our study include analyzing a patient-oriented outcome endpoint in evaluating epinephrine treatment effects from a long term perspective, and create equipoise about the current standard of resuscitation care. After employing strict statistical adjustment to residual confounders, the findings of this observational study are consistent with previous studies, and can provide more evidence and inspire further investigation on the most appropriate scheme of CA emergency treatment.

This study is limited by its observational nature, failing to establish causal relationships between epinephrine dosing and neurological outcomes and is prone to “resuscitation bias,” where the estimates of intra-cardiac arrest interventions will be biased toward a harmful effect (Andersen et al., 2018). Also, the research subjects were comatose patients (Glasgow Coma Scale, GCS <9) admitted after IHCA or OHCA from Department of Intensive Care at Erasme Hospital, Brussels (Belgium). Therefore, there is a certain deficiency in the universality and extrapolation of research. As a secondary analysis, we had limited access to sample size and detailed information, such as quality or duration of CPR, which are important variables when assessing intra-cardiac arrest interventions (Matos et al., 2013; Wengenmayer et al., 2017). Nevertheless, such covariates can be tricky to quantify under specific circumstances, and are also absent from previous studies investigating the impact of epinephrine dose on cardiac arrest survival and neurological outcomes (Callaway, 2013; Dumas et al., 2014; Tujjar et al., 2015). Further studies are therefore needed to validate the impact of total epinephrine dose on long-term neurological outcomes.

## Conclusion

In this cohort of patients who underwent OHCA or IHCA, we observed an adverse association between total epinephrine dosing during resuscitation and neurological status three months after the event, after adjusting other confounding factors. This negative effect of epinephrine was not eliminated by targeted temperature management (TTM). These finding suggest that further researches are needed to investigate the long-term effect of epinephrine on cardiac arrest patients.

## Data Availability

All data generated or analyzed during this study are included in this article/Supplementary Material, further inquiries can be directed to the corresponding author/s.
