# Supplementary material for: Impact of Total Epinephrine Dose on Long Term Neurological Outcome for Cardiac Arrest Patients: A Cohort Study
Source: Front Pharmacol. 2021 May 28;12:580234. doi: 10.3389/fphar.2021.580234 (PMC8193671; doi:10.3389/fphar.2021.580234)
Supplement: Supplementary file 1 [file DataSheet1.pdf]

Appendix data

Impact of Total Epinephrine Dose on Long Term Neurological Outcome for Cardiac Arrest Patients: A Cohort Study

Appendix Table 1. Coefficient of each variable in univariate and multivariate model.

Exposure	Univariate (OR,95%CI, P value)	Multivariate (OR,95%CI, P value)
Age (year)	0.98 (0.97, 0.99) 0.005	0.98 (0.96, 0.99) 0.003
Epinephrine dosage (mg)		
< 2 mg	Reference	Reference
2 mg	0.84 (0.44, 1.61) 0.600	0.80 (0.3834, 1.68) 0.561
3-4 mg	0.45 (0.25, 0.81) 0.008	0.43 (0.21, 0.89) 0.024
≥5 mg	0.32 (0.18, 0.56) <0.001	0.40 (0.17, 0.96) 0.041
Gender (male)	1.14 (0.71, 1.81) 0.593	1.24 (0.72, 2.16) 0.441
Previous neurological disease	0.43 (0.22, 0.84) 0.013	0.38 (0.18, 0.82) 0.014
Chronic renal failure	0.74 (0.42, 1.32) 0.307	0.83 (0.42, 1.66) 0.602
OHCA	0.98 (0.65, 1.49) 0.940	1.23 (0.66, 2.27) 0.511
Witnessed arrest	2.60 (1.32, 5.13) 0.006	1.85 (0.84, 4.09) 0.129
Bystander CPR	2.07 (1.30, 3.31) 0.002	1.7536 (0.96, 3.19) 0.065
Time to ROSC (min)	0.97 (0.96, 0.99) <0.001	0.98 (0.95, 1.004) 0.094
Non-shockable rhythm	0.29 (0.19, 0.45) <0.001	0.30 (0.17, 0.50) <0.001
TTM	0.56 (0.29, 1.06) 0.077	0.66 (0.31, 1.41) 0.282
ICU stay (day)	1.03 (1.00, 1.05) 0.027	1.03 (1.00, 1.05) 0.020
Baseline glucose (mEq l-1)	1.0021 (1.0003, 1.0038) 0.020	1.0015 (0.9995, 1.0035) 0.130
Baseline lactate (mEq l-1)	0.96 (0.90, 1.03) 0.229	0.9504 (0.88, 1.03) 0.191

Abbreviation: OHCA: out-of-hospital cardiac arrest; CPR, cardiopulmonary

resuscitation; ROSC, restoration of spontaneous circulation; TTM, targeted temperature management; ICU, intensive care unit

Appendix Table 2. Assumption check for multivariate logistic regression model.

	**Estimate**	**Std. Error**	**z value**	**exp(coef)**	**95% CI**	**P value**
Intercept	1.67	0.96	1.74	5.34	0.81, 35.23	0.082
Age (year)	-0.02	0.01	-2.79	0.98	0.96, 0.99	0.005
Epinephrine dosage 2 mg	-0.22	0.38	-0.58	0.80	0.38, 1.68	0.561
Epinephrine dosage 3-4 mg	-0.84	0.37	-2.26	0.43	0.21, 0.89	0.024
Epinephrine dosage ≥5 mg	-0.91	0.45	-2.04	0.40	0.17, 0.96	0.041
Male	0.22	0.28	0.77	1.24	0.72, 2.16	0.441
Previous neurological disease	-0.96	0.39	-2.45	0.38	0.18, 0.82	0.014
Witnessed arrest	0.61	0.40	1.52	1.85	0.84, 4.08729	0.129
TTM	-0.42	0.39	-1.08	0.66	0.31, 1.41	0.282
ICU length of stay	0.03	0.01	2.33	1.03	1.00, 1.05	0.020
Non-shockable rhythm	-1.22	0.27	-4.56	0.30	0.17, 0.50	<0.001
Baseline glucose (mEq l-1)	0.002	0.001	1.51	1.002	0.999, 1.004	0.130
Time to ROSC	-0.02	0.01	-1.67	0.98	0.953, 1.004	0.094
By stander CPR	0.56	0.30	1.84	1.75	0.96, 3.19	0.065
Chronic renal failure	-0.18	0.35	-0.52	0.83	0.42, 1.66	0.602
OHCA	0.21	0.31	0.66	1.23	0.66, 2.27	0.511
Baseline lactate (mEq l-1)	-0.05	0.04	-1.31	0.95	0.88, 1.03	0.191

AIC: 441.5755; Log Likelihood: -203.7878, df= 18

Abbreviation: TTM, targeted temperature management; ICU, intensive care unit; ROSC, restoration of spontaneous circulation; CPR, cardiopulmonary resuscitation; OHCA: out-of-hospital cardiac arrest

Appendix Table 3. Effect values of variables (age and baseline lactate) that are in curvilinear relationships with the outcome are further checked by generalized addictive model.

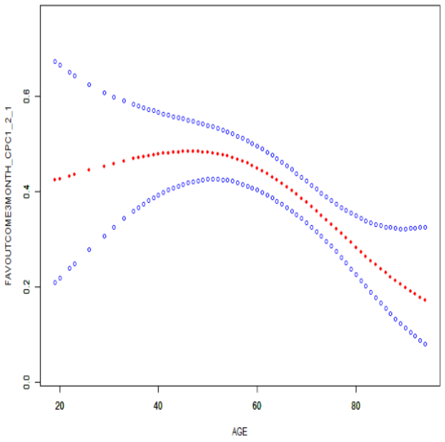

	Estimate	Std. Error	z value	Pr(>|z|)	exp(est)	95% CI
Intercept	-0.4362	0.1079	-4.041	1.00E-04	0.6465	(0.5232,0.7988)

Approximate significance of smooth terms

	edf	Ref. df	Chi. sq	P value
[a] **s(Age)**	2.0909	2.6551	11.897	0.0065

[a] s(Age) indicates that age is adjusted by generalized addictive model as a curvilinear variable.

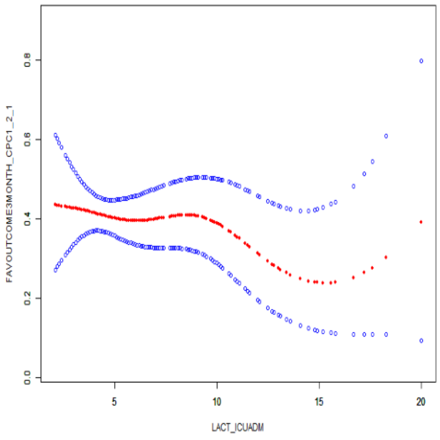

	Estimate	Std. Error	z value	Pr(>|z|)	exp(est)	95% CI
Intercept	-0.423	0.1063	-3.9793	1.00E-04	0.6551	(0.5319,0.8068)

Approximate significance of smooth terms

	edf	Ref. df	Chi. sq	P value
[a] **s(Baseline lactate)**	3.4395	4.2888	2.8126	0.5584

[a] s(Baseline lactate) indicates that age is adjusted by generalized addictive model as a curvilinear variable.

Appendix Table 4. The effect of epinephrine dosage on 3-month neurological outcomes is consistent among OHCA/IHCA groups and shockable/non-shockable rhythm groups, as revealed by stratification analysis.

	OR, 95%CI, P value				OR, 95%CI, P value			
**Exposure**	**OHCA**	**IHCA**	**Total**	**p interaction**	**Shockable rhythm**	**Non-shockable rhythm**	**Total**	**p interaction**
**Non-adjusted**	0.84<br>(0.75, 0.96)<br>0.007	0.86<br>(0.79, 0.94)<br>0.001	0.86<br>(0.80, 0.92)<br><0.001	0.798	0.89 (0.80,0.98)<br>0.023	0.82<br>(0.73, 0.92)<br><0.001	0.85<br>(0.79, 0.92)<br><0.001	0.321
**Adjust I**	0.81<br>(0.71, 0.93)<br>0.003	0.84<br>(0.77, 0.93)<br>0.001	0.83<br>(0.77, 0.90)<br><0.001	0.602	0.87<br>(0.77, 0.97)<br>0.012	0.80<br>(0.71, 0.90)<br><0.001	0.83<br>(0.77, 0.90)<br><0.001	0.213
**Adjust II**	0.72<br>(0.56, 0.93)<br>0.012	0.91<br>(0.79, 1.05)<br>0.213	0.86<br>(0.76, 0.98)<br>0.019	0.425	0.87<br>(0.73, 1.05)<br>0.140	0.84<br>(0.70, 1.01)<br>0.064	0.86<br>(0.76, 0.98)<br>0.019	0.190

Abbreviation: OHCA, out-of-hospital cardiac arrest; IHCA, in-hospital cardiac arrest

Appendix Table 5. Demographic data of OHCA and IHCA patients.

**Variable**	**IHCA （N=166）**	**OHCA (N=207)**	**P-value**
Age (year)	63.36 ± 15.60	60.59 ±15.17	0.085
Time to ROSC (min)	14.02 ±13.31	21.22 ±13.91	<0.001
Epinephrine dosage (mg)	3.54 ±3.48	4.45 ±3.79	0.017
Baseline glucose(mg dl^{-1})	6.50 ±3.56	6.05 ±3.11	0.194
Baseline lactate (mEq l^{-1})	243.73 ±117.04	227.42 ±131.27	0.212
ICU stay (day)	9.67 ±11.60	6.58 ±7.66	0.002
Gender (male)	115 (69.28%)	154 (74.40%)	0.273
Hypertension	81 (48.80%)	77 (37.20%)	0.024
Diabetes	54 (32.53%)	36 (17.39%)	<0.001
Chronic heart failure	50 (30.12%)	28 (13.53%)	<0.001
Chronic renal failure	48 (29.09%)	14 (6.76%)	<0.001
Previous neurological disease	24 (14.46%)	30 (14.49%)	0.992
Bystander CPR	149 (89.76%)	105 (50.72%)	<0.001
Witnessed arrest	157 (94.58%)	162 (78.26%)	<0.001
Non-shockable rhythm	123 (74.10%)	97 (46.86%)	<0.001
TTM	134 (81.21%)	196 (94.69%)	<0.001
ICU death	81 (48.80%)	112 (54.11%)	0.308
Hospital death	96 (57.83%)	116 (56.04%)	0.728
**Favorable neurological outcomes**	66 (39.76%)	82 (39.61%)	0.977

Abbreviation: ROSC, restoration of spontaneous circulation; CPR, cardiopulmonary resuscitation; TTM, targeted temperature management; ICU, intensive care unit;
